# Is involvement in school bullying associated with increased risk of murderous ideation and behaviours among adolescent students in China?

**DOI:** 10.1186/s12888-019-2108-5

**Published:** 2019-04-24

**Authors:** Pu-Yu Su, Geng-Fu Wang, Huan He, A-Zhu Han, Guo-Bao Zhang, Nuo Xu

**Affiliations:** 1 Department of Maternal, Child and Adolescent Health, School of Public Health, Anhui Medical University, No. 81 Meishan road, Hefei, 230032 Anhui China; 2 Anhui Provincial Key Laboratory of Population Health and Aristogenics, Hefei, Anhui China; 3 Department of Maternal, Child and Adolescent Health, School of Public Health, Tianjin Medical University, No. 22 Qixiangtai Road, Tianjin, 300070 China; 4 Institute of Population, Family and Reproductive Health, Johns Hopkins Bloomberg School of Public Health, Baltimore, MD USA

**Keywords:** Adolescent, School bullying, Murderous ideation and behaviours

## Abstract

**Background:**

School bullying is a destructive behaviour common among adolescents that can sometimes escalate to criminal activity. This study aimed to examine the association between four types of school bullying (i.e., physical, verbal, relational, and cyber) and murderous ideation and behaviours (i.e., ideation, plans, preparation, and attempts) among adolescent students.

**Methods:**

Data were collected from 5726 middle and high school students using self-administered questionnaires in December 2013. The participants were selected using a 3-stage random cluster-sampling strategy. The participants were asked about the frequency of their bullying experiences in the past two months and the frequencies of their murderous ideation and behaviours in the past six months. Multivariate logistic regressions were performed to explore the association between school bullying and murderous ideation and behaviours.

**Results:**

Each type of school bullying perpetration was associated with murderous ideation and behaviours, as was each type of bullying victimization. Students who experienced more types of school bullying perpetration and victimization were more likely to report murderous ideation and behaviours. Moreover, the number of types of bullying perpetration and victimization had a dose-response association with murderous ideation and behaviours (aOR min = 1.45, aOR max = 2.72), as did the frequency of involvement in bullying perpetration and victimization (aOR min = 1.33, aOR max = 2.00). Being a bully-victim was a risk factor for murderous ideation and behaviours (aOR min = 3.88, aOR max = 7.24).

**Conclusions:**

Each type of school bullying was associated with an increased risk for murderous ideation and behaviours among adolescents. Dose-response relationships between the frequency of bullying and number of bullying types experienced and murderous ideation and behaviours were found in this study. Future studies are warranted to confirm our findings and explore the mechanisms underlying the relationship between school bullying and murderous ideation and behaviours.

**Electronic supplementary material:**

The online version of this article (10.1186/s12888-019-2108-5) contains supplementary material, which is available to authorized users.

## Background

Adolescent homicide has received public attention in the last decade. In the United States, in 2010, the homicide rate is 11.7 per 100,000 people among youths aged 15–24 years, which is significantly higher than the homicide rate among the general population (4.8 per 100,000 population) [1]. Approximately one-third of homicides were perpetrated by victims’ peers, and these incidents should be considered murder [2]. Data from the National Bureau of Statistics of China showed that there were a total of 55,817 juvenile crime cases in 2013 [3]. In 2013, a study by Lu et al. [4] surveyed a nationally representative sample including 990 juvenile offenders and revealed that 2/3 of juvenile offenders were 14 to 16 years old, and 6.6% of juvenile offenders had committed the crime of intentional homicide. The impact of adolescent murder can be profound and long lasting [5]. Murders can cause the deaths of victims and have a negative impact on surviving victims, including mental health problems and physical disability. Moreover, homicide survivors are more likely to report depression, drug abuse, and alcohol abuse [6]. Exposure to a homicide has an acute negative effect on the cognitive performance of children across a neighbourhood [7].

School bullying is defined as repeated, negative acts that are committed by one or more adolescents against another [8]. School bullying can be divided into four types: physical, verbal, relational, and cyber [9]. Modecki et al. [10] reviewed the literature on adolescent school bullying across different contexts and reported that the mean prevalence rates of traditional bullying (i.e., physical, verbal and relational) and cyber bullying involvement were 35 and 15%, respectively. Studies conducted in mainland China have shown that the prevalence rates of self-reported bullying victimization and perpetration range from 2 to 66% and 2 to 34%, respectively, whereas the prevalence rates of self-reported cyber bullying victimization and perpetration range from 14 to 57% and 3 to 35%, respectively [11]. Emerging evidence indicates that involvement in bullying is associated with physical health problems, behavioural problems, and even suicidal ideation and behaviours [12–17].

A growing body of studies has reported the associations between school bullying and adolescent violent behaviours, including physical violence, teen dating violence and suicide [18–20]. School bullying was previously found to be a predictor of violence in later life [21]. Further, studies have indicated that homicide may be caused by school bullying [22, 23]. Involvement in bullying as a victim, bully, or bully-victim is associated with weapon carrying, which increases the likelihood of death during conflict issues [24]. Previous studies have found that adolescents involved in bullying shared several risk factors for committing homicide. For example, adverse childhood experiences, such as childhood maltreatment, have been reported as a risk factor for both bullying and homicide [25, 26]. Additionally, adolescents involved in bullying may have mental health or neurodevelopmental problems that make them susceptible to homicide. For example, elevated and uncontrolled anger/aggression can increase the risk of homicide/attempted homicide [27]. Camodeca and Goossens [28] found that both bullies and victims scored higher in hostile aggression, anger, retaliation, and ease of aggression. Similarly, adolescents involved in bullying were associated with increased risk for drug and alcohol abuse and mental disorders; thus, they were more likely to commit homicide [12–16, 26, 29, 30]. However, few studies have examined the relationship between school bullying and adolescent murder.

In this study, we hypothesized that school bullying may be significantly associated with murderous ideation and behaviours. First, we aimed to study the association between each type of school bullying (including traditional and cyber bullying) and murderous ideation and behaviours. A previous study found that the impact of cyber bullying on victims is comparable to that of traditional bullying on victims [31]. Therefore, it is hypothesized that involvement in both traditional bullying and cyber bullying is associated with an increased risk for adolescent murderous ideation and behaviours. Second, we further examined the association between the frequency of school bullying and number of bullying types and murderous ideation and behaviours, as previous studies have shown that depression and poor sleep quality occurred more frequently after experiencing multiple types of school bullying [32, 33]. Additionally, we hypothesized that there is a dose-response relationship between the frequency of school bullying and number of bullying types and murderous ideation and behaviours. Third, we aimed to investigate the relationship between the role played in school bullying and murderous ideation and behaviours. We hypothesized that involvement in school bullying as a victim, bully, or bully-victim is related to murderous ideation and behaviours. This study can provide scientific evidence for further research and efforts to prevent murder among adolescents in China.

## Methods

### Study design and participants

This study was part of the research project “Adolescent Health and Risky Behaviours in Anhui Province”. Detailed information regarding this study has been described previously [25, 34, 35]. The study protocol was reviewed and approved by the Biomedicine Ethics Committee of Anhui Medical University. Briefly, a 3-stage random cluster-sampling approach was employed to select participants in Anhui province, an area in the middle of China. We got a total effective sample of 5726 middle and high school students, including 2848 boys and 2878 girls. The students ranged from 12 to 18 years old, and the mean (±SD) age was 14.81 ± 1.96 years.

### Measures

#### Murderous ideation and Behaviours

In this study, we considered murder as a series of psychological and behaviour processes encompassing ideation, plans, preparation, and attempts, whereas homicide was the outcome of causing someone’s death. Four items employed to measure adolescents’ murderous ideation and behaviours (i.e., ideation, plans, preparation, and attempts) in the past six months, which have been described in our previous study [35]. Each item was coded as a dichotomous variable: yes vs. no.

Before conducting the survey, the same questionnaire was tested among 156 middle and high school students to ensure that our study participants could understand the survey questions and complete the survey independently. The items assessing murderous ideation and behaviours showed desirable reliability (the Kappa values ranged from 0.81 to 0.87; Cronbach’s alphas ranged from 0.79 to 0.92).

#### Bullying experiences

The school bullying questions were adopted or modified from previous studies [9, 36], except for four new items we created based on our local social context. In the questionnaire, we provided a standard definition of bullying (qifu) in Chinese [37]. A total of sixteen items were used to measure experiences of bullying perpetration and victimization regarding four types of school bullying (i.e., physical, verbal, relational, and cyber) in the past two months. Detailed information about those specific items has been described in our previous study [25].

In the data analysis, each type of bullying was coded as a dichotomous variable (yes vs. no). To compare the prevalence of different forms of bullying in alignment with a previous study [13], a more conservative criterion (two to three times a month or more) was used to measure traditional and cyber bullying. To investigate the dose-response relationship between school bullying and murderous ideation and behaviours, bullying experiences were also measured as two ordinary variables: the number of bullying types one perpetrated (i.e., not involved, one type, two types, three types, or four types) and the frequency of perpetration (i.e., none, less than twice a month, two or three times per month, once a week, or multiple times per week). Similarly, victimization experiences were measured as two ordinary variables: the number of bullying victimization types one experienced (i.e., not involved, one type, two types, three types, or four types) and the frequency of victimization (i.e., none, less than twice a month, two or three times per month, once a week, or multiple times per week). Moreover, the students were divided into four groups (not involved, bully only, victim only, and bully-victim) based on their bullying experiences, as previous study described [25].

Our study results reached a high level of agreement (Kappa coefficient) between the early and later (an interval of a week) responses to each bullying questionnaire, ranging from 0.85 to 0.96. Moreover, the internal consistency of each bullying questionnaire also reached a high level of agreement, ranging from 0.74 to 0.80.

#### Covariates

Based on our previous studies [25, 35], the covariates included gender, grade, self-estimated family economic status, relationship with parents, and number of friends.

### Statistical analysis

The distribution of covariates in different outcome groups was evaluated using odds ratios (ORs), and ORs were calculated for covariates and study outcomes (i.e., ideation, plans, preparation, and attempts). Multivariate logistic regressions containing a single predictor were performed to explore the association between school bullying and murderous ideation and behaviours with the adjustment of covariates. Each type of school bullying perpetration (yes vs. no) and victimization (yes vs. no), the number of perpetration and victimization types one was involved in (categorical variable or continuous variable), the frequency of perpetration or victimization (categorical variable or continuous variable) and role in school bullying were coded as predictors in each single predictor model. Adjusted ORs (aORs) were calculated to adjust the covariates in the multivariate logistic regression model. All statistical analyses were performed using SPSS 23.0 (SPSS, Inc., Chicago, IL, USA). We also fit two-level logistic regression models in which classrooms were treated as clusters using the package “lme4” in R version 3.5.1. The results remained robust. We included those results in the Supplemental Materials (Additional file 1: Table S1, Additional file 2: Table S2, Additional file 3: Table S3 and Additional file 4: Table S4).

## Results

As shown in Table 1, the prevalence rates of murderous ideation, plans, preparation, and attempts among our study participants were 9.9, 2.8, 1.3, and 0.6%, respectively. The characteristics of the study participants were associated with individual types of murderous ideation and behaviours at varying degrees. Males were more likely than females to report all four types of study outcomes. Adolescents with poor self-estimated family economic status were more likely to report murderous ideation than adolescents with general self-estimated family economic status. Having a better self-reported relationship with parents was associated with lower ORs for both murderous ideation and plans [mother to ideation: 0.60(0.50 to 0.72), mother to plans: 0.51(0.37 to 0.71), father to ideation: 0.53(0.44 to 0.63), father to plans: 0.63(0.46 to 0.86)].

**Table 1:** Unadjusted odds ratios (with 95% CI) for murderous ideation and behaviours (*N* = 5726)

Category	%	Ideation		Plans		Preparation		Attempts	
		%	OR (95%CI)	%	OR (95%CI)	%	OR (95%CI)	%	OR (95%CI)
Total	100.0	9.9		2.8		1.3		0.6	
Gender									
Male	49.7	12.2	1.00 [Ref]	3.9	1.00 [Ref]	2.1	1.00 [Ref]	1.0	1.00 [Ref]
Female	50.3	7.7	**0.60 (0.51 to 0.72)**	1.6	**0.41 (0.29 to 0.57)**	0.6	**0.28 (0.16 to 0.48)**	0.1	**0.14 (0.05 to 0.40)**
Grade									
Grade 7 to 9	51.7	9.9	1.00 [Ref]	3.1	1.00 [Ref]	1.4	1.00 [Ref]	0.6	1.00 [Ref]
Grade 10 to 12	48.3	10.0	1.02 (0.85 to 1.21)	2.4	0.77 (0.56 to 1.06)	1.2	0.86 (0.55 to 1.36)	0.5	0.73 (0.36 to 1.48)
Self-estimated family economic status									
Poor	9.6	12.9	1.00 [Ref]	3.6	1.00 [Ref]	1.0	1.00 [Ref]	0.5	1.00 [Ref]
General	76.4	9.2	**0.68 (0.54 to 0.86)**	2.6	0.71 (0.47 to 1.07)	1.4	1.40 (0.67 to 2.94)	0.6	1.24 (0.43 to 3.54)
Good	14.0	11.7	0.89 (0.64 to 1.25)	3.1	0.85 (0.46 to 1.56)	1.3	1.28 (0.46 to 3.55)	0.2	0.36 (0.40 to 3.26)
Family structure									
Nuclear family	66.2	9.8	1.00[Ref]	2.9	1.00[Ref]	1.5	1.00[Ref]	0.6	1.00[Ref]
Big family	23.6	10.3	1.06 (0.86 to 1.30)	2.5	0.87 (0.59 to 1.29)	1.0	0.69 (0.38 to 1.23)	0.5	0.82 (0.35 to 1.90)
Single-parent family	8.2	11.1	1.15 (0.85 to 1.57)	3.2	1.12 (0.65 to 1.93)	0.9	0.56 (0.20 to 1.56)	0.2	0.34 (0.05 to 2.49)
Others	2.0	7.1	0.71 (0.34 to 1.47)	0.9	0.30 (0.04 to 2.20)	0.9	0.59 (0.08 to 4.30)	0.0	NA
Relationship with mother									
Poor	23.6	13.8	1.00 [Ref]	4.4	1.00 [Ref]	1.6	1.00 [Ref]	0.8	1.00 [Ref]
Good	76.4	8.7	**0.60 (0.50 to 0.72)**	2.3	**0.51 (0.37 to 0.71)**	1.2	0.76 (0.46 to 1.25)	0.5	0.59 (0.28 to 1.22)
Relationship with father									
Poor	33.4	14.0	1.00 [Ref]	3.7	1.00 [Ref]	1.6	1.00 [Ref]	0.7	1.00 [Ref]
Good	66.6	7.9	**0.53 (0.44 to 0.63)**	2.3	**0.63 (0.46 to 0.86)**	1.2	0.77 (0.48 to 1.22)	0.5	0.73 (0.36 to 1.48)
Number of friends									
< 3	25.1	12.4	1.00 [Ref]	3.6	1.00 [Ref]	0.8	1.00 [Ref]	0.2	1.00 [Ref]
≥ 3	74.9	9.1	**0.70 (0.58 to 0.85)**	2.8	**0.68 (0.49 to 0.96)**	1.5	**1.99 (1.05 to 3.80)**	0.7	3.26 (0.99 to 10.72)

Note: % refers to percent of positive ideation, plans, preparation and attempts in each demographic category. OR - odds ratios; CI - confidence interval

Variable levels significant at *p* < 0.05 are in boldface type

### Traditional bullying or cyberbullying and murderous ideation and Behaviours

As shown in Table 2, both those who perpetrated bullying and those who were bullied had higher ORs for murderous ideation, plans, preparation, and attempts than those who did not have relevant experiences. The ORs in Table 2 are all statistically significant. For those who committed bullying, the ORs for the study outcomes increased gradually from ideation to plans, preparation, and then attempts. This trend was consistently found among participants who committed any of the four bullying types. However, for those who were bullied, this trend was observed only for cyber bullying. Cyber bullying was found to be highly associated with murderous attempts in both bullies [25.69 (12.31 to 53.59)] and bullying victims [23.90 (11.54 to 49.51)].

**Table 2:** Multivariate logistic regression between individual type of school bullying and adolescent murderous ideation and behaviours (*N* = 5726)

Type of school bullying experience	%	Ideation		Plans		Preparation		Attempts	
		%	aOR (95%CI)^a^	%	aOR (95%CI)^b^	%	aOR (95%CI)^c^	%	aOR (95%CI)^d^
Bully									
Physical	3.5	23.2	**2.43 (1.72 to 3.45)**	15.2	**5.97 (3.86 to 9.23)**	9.6	**7.98 (4.60 to 13.86)**	6.1	**12.79 (6.09 to 26.86)**
Verbal	6.6	24.2	**2.84 (2.19 to 3.68)**	11.1	**4.51 (3.09 to 6.59)**	6.1	**5.05 (3.03 to 8.43)**	3.9	**9.23 (4.51 to 18.90)**
Relational	3.8	25.8	**3.13 (2.26 to 4.31)**	16.6	**7.72 (5.14 to 11.59)**	10.6	**10.74 (6.40 to 18.01)**	5.1	**11.38 (5.38 to 24.08)**
Cyber	2.4	24.3	**2.60 (1.72 to 3.91)**	17.6	**7.00 (4.32 to 11.35)**	14.7	**13.35 (7.66 to 23.28)**	10.3	**25.69 (12.31 to 53.59)**
Victim									
Physical	6.1	20.5	**2.09 (1.58 to 2.78)**	8.4	**2.79 (1.81 to 4.27)**	4.6	**2.93 (1.91 to 4.49)**	3.2	**5.87 (2.78 to 12.42)**
Verbal	15.7	15.9	**1.67 (1.35 to 2.06)**	5.7	**2.09 (1.47 to 2.96)**	2.9	**2.19 (1.55 to 3.10)**	1.6	**3.17 (1.56 to 6.44)**
Relational	10.8	19.7	**2.34 (1.87 to 2.93)**	7.3	**3.12 (2.18 to 4.48)**	3.6	**3.27 (2.28 to 4.68)**	1.8	**4.21 (2.01 to 8.79)**
Cyber	2.7	22.7	**2.47 (1.67 to 3.66)**	14.3	**5.62 (3.44 to 9.16)**	9.1	**5.63 (3.45 to 9,16)**	9.1	**23.90 (11.54 to 49.51)**

Note: % refers to percent of positive ideation, plans, preparation and attempts in each type of school bullying experience

^a^ Model adjusted for gender, self-estimated family economic status, relationship with mother, relationship with father and number of friends that were statistically significant in univariate analyses

^b^ Model adjusted for gender, relationship with mother, relationship with father and number of friends

^c^ Model adjusted for gender and number of friends

^d^ Model adjusted for gender

*aOR* Adjusted odds ratios; *CI* Confidence interval

Results arrived statistically significant at *p* < 0.05 are in boldface type

### Number of bullying types and murderous ideation and Behaviours

Table 3 shows the associations between the number of bullying types and adolescent murder. We divided the participants into five groups (i.e., not involved, one type, two types, three types, and four types) by the number of types of bullying they committed or experienced. Compared with those who were not involved school bullying perpetration, participants who committed all four types of school bullying were more likely to report murderous ideation [4.49 (2.38–8.45)], plans [18.37(9.39–36.97)], preparation [28.38(12.97–62.10)], and attempts [59.51 (22.90–154.69)]. In addition, the ORs increased with the number of bullying types perpetrated. Correspondently, participants who were the victims of all four types of bullying showed significantly higher ORs for all four types of murderous ideation and behaviours. The ORs for murder-related outcomes increased with the number of bullying types experienced except for murderous ideation. Of the four studied murder-related outcomes, “ideation” had the lowest ORs, and “attempts” had the highest ORs.

**Table 3:** Multivariate logistic regression of adolescent murderous ideation and behaviours on number of school bullying types (*N* = 5726)

Variety of school bullying experience	%	Ideation		Plans		Preparation		Attempts	
		%	aOR (95%CI) ^a^	%	aOR (95%CI) ^b^	%	aOR (95%CI) ^c^	%	aOR (95%CI) ^d^
Bully									
Non-involved	76.7	8.4	1.00 [Ref]	1.9	1.00 [Ref]	0.8	1.00 [Ref]	0.3	1.00 [Ref]
One type	15.2	22.5	**2.83 (2.17 to 3.69)**	7.1	**3.34 (2.14 to 5.22)**	3.1	**3.49 (1.81 to 6.73)**	1.3	**4.21 (1.48 to 11.94)**
Two types	5.0	22.0	**2.65 (1.66 to 4.25)**	10.1	**4.57 (2.35 to 8.89)**	5.5	**5.66 (2.32 to 13.80)**	2.8	**7.74 (2.15 to 27.91)**
Three types	2.2	22.4	**2.79 (1.40 to 5.56)**	18.4	**9.91 (4.61 to 21.28)**	14.3	**15.60 (6.53 to 37.29)**	6.1	**17.91 (4.87 to 65.91)**
Four types	0.9	32.6	**4.49 (2.38 to 8.45)**	30.4	**18.37 (9.39 to 36.97)**	21.7	**28.38 (12.97 to 62.10)**	17.4	**59.51 (22.90 to 154.69)**
Number of types			**1.59 (1.43 to 1.77)**		**2.12 (1.85 to 2.43)**		**2.36 (2.00 to 2.78)**		**2.72 (2.18 to 3.39)**
Victim									
Non-involved	89.7	8.0	1.00 [Ref]	2.0	1.00 [Ref]	0.9	1.00 [Ref]	0.3	1.00 [Ref]
One type	6.7	13.4	**1.60 (1.28 to 2.01)**	3.4	**1.54 (1.01 to 2.36)**	1.5	1.50 (0.80 to 2.83)	0.6	1.80 (0.63 to 5.13)
Two types	1.9	19.9	**2.44 (1.78 to 3.35)**	7.0	**3.04 (1.82 to 5.05)**	3.1	**3.12 (1.49 to 6.54)**	1.4	**4.05 (1.29 to 12.71)**
Three types	0.9	25.8	**3.28 (2.14 to 5.02)**	12.1	**5.09 (2.82 to 9.21)**	7.3	**7.00 (3.28 to 14.97)**	5.6	**15.66 (5.98 to 40.99)**
Four types	0.8	21.6	**2.64 (1.33 to 5.24)**	15.7	**7.49 (3.83 to 16.60)**	9.8	**9.28 (3.45 to 24.95)**	7.8	**21.58 (6.62 to 70.36)**
Number of types			**1.45 (1.32 to 1.59)**		**1.69 (1.48 to 1.94)**		**1.81 (1.51 to 2.18)**		**2.29 (1.79 to 2.93)**

Note: % refers to percent of positive ideation, plans, preparation and attempts in each type of school bullying experience

^a^ Model adjusted for gender, self-estimated family economic status, relationship with mother, relationship with father and number of friends that were statistically significant in univariate analyses

^b^ Model adjusted for gender, relationship with mother, relationship with father and number of friends

^c^ Model adjusted for gender and number of friends

^d^ Model adjusted for gender

*aOR* Adjusted odds ratios,*CI* Confidence interval

Results arrived statistically significant at *p* < 0.05 are in boldface type

In addition to the ORs reported above, we further examined the dose-response relationship between the number of bullying types and murder-related outcomes by inputting the number of bullying types in which a participant was involved as continuous variables in the logistic regression. The results are shown in Table 3. For those who committed bullying, committing one more type of bullying statistically significantly increased the ORs for murderous ideation [1.59 (1.43 to 1.77)], plans [2.12(1.85 to 2.43)], preparation [2.36 (2.00 to 2.78)], and attempts [2.72 (2.18 to 3.39)]. For those who were bullied, experiencing one more type of bullying also significantly increased the ORs for murderous ideation [1.45 (1.32 to 1.59)], plans [1.69 (1.48 to 1.94)], preparation [1.81 (1.51 to 2.18)], and attempts [2.29 (1.79 to 2.93)]. The magnitude of the effect increased gradually from murderous ideation to attempts, and the difference between every two types of murder-related outcomes was higher than 10%.

### Frequency of involvement in school bullying and murderous ideation and Behaviours

Table 4 shows the association between the frequency of bullying and the murder-related outcomes. For both bullies and victims, being more frequently involved in school bullying was associated with a higher risk for murderous ideation, plans, preparation, and attempts. In the logistic regression analysis, we categorized the study participants into five subgroups (i.e., not involved, less than twice a month, two or three times a month, once a week, or multiple times per week) based on the frequency of their bullying experiences. In the results, for both bullies and victims, all subgroups with bullying experience were significantly associated with higher odds of murderous ideation compared to those who had no bully related experience. Only the higher frequencies (i.e., once or more than once a week) of bullying experience were significantly associated with murderous plans and murderous preparation. Murderous attempts were significantly associated with the subgroup that reported multiple experiences of bullying per week. We additionally examined the dose-response relationship by using a continuous variable for bullying frequency in the logistic regression model. The results showed that the frequencies of bullying experience were significantly associated with all four types of murder-related outcomes. The ORs were highest for murderous attempts [bully: 2.00 (1.59 to 2.51), victim: 1.53(1.22 to 1.93)], followed by preparation [bully: 1.82 (1.57 to 2.11), victim: 1.43 (1.23 to 1.65)], plans [bully: 1.64(1.48 to 1.82), victim: 1.36 (1.23 to 1.51)], and ideation [bully: 1.49 (1.40 to 1.58), victim: 1.33(1.25 to 1.41)].

**Table 4:** Multivariate logistic regression of adolescent murderous ideation and behaviours on frequency of school bullying (*N* = 5726)

Frequency of school bullying	%	Ideation		Plans		Preparation		Attempts	
		%	aOR (95% CI)^a^	%	aOR (95% CI)^b^	%	aOR (95% CI) ^c^	%	aOR (95% CI) ^d^
Bully									
Non-involved	62.3	6.3	1 [Reference]	1.7	1 [Reference]	0.7	1 [Reference]	0.3	1 [Reference]
Less than twice a month	19.3	11.0	**1.76 (1.39 to 2.22)**	1.9	1.04 (0.63 to 1.72)	0.6	0.79 (0.34 to 1.82)	0.1	0.25 (0.03 to 1.97)
Two or three times a month	8.1	16.5	**2.72 (2.05 to 3.61)**	3.2	1.68 (0.94 to 3.00)	1.3	1.55 (0.63 to 3.80)	0.2	0.58 (0.07 to 4.47)
Once a week	4.8	26.2	**4.70 (3.46 to 6.38)**	6.9	**3.59 (2.10 to 6.15)**	3.6	**4.32 (2.05 to 9.14)**	0.7	1.81 (0.40 to 8.27)
Multiple times per week	5.5	23.2	**3.92 (2.90 to 5.31)**	13.7	**7.26 (4.76 to 11.09)**	8.6	**9.79 (5.23 to 17.33)**	5.4	**12.45 (5.65 to 27.41)**
Score of frequency			**1.49 (1.40 to 1.58)**		**1.64 (1.48 to 1.82)**		**1.82 (1.57 to 2.11)**		**2.00 (1.59 to 2.51)**
Victim									
Non-involved	42.1	6.2	1 [Reference]	1.7	1 [Reference]	0.9	1 [Reference]	0.5	1 [Reference]
Less than twice a month	22.6	8.3	**1.31 (1.01 to 1.69)**	1.9	1.05 (0.64 to 1.73)	0.8	0.85 (0.40 to 1.81)	0.0	NA
Two or three times a month	13.2	12.1	**1.94 (1.47 to 2.56)**	2.4	1.24 (0.71 to 2.18)	0.9	0.99 (0.42 to 2.34)	0.1	0.26 (0.03 to 1.98)
Once a week or more	8.4	13.8	**2.11 (1.55 to 2.89)**	3.8	**1.82 (1.03 to 3.21)**	1.7	1.70 (0.74 to 3.87)	0.6	1.10 (0.31 to 3.98)
Multiple times per week	13.8	19.7	**3.15 (2.46 to 4.03)**	7.1	**3.37 (2.22 to 5.11)**	3.8	**3.80 (2.14 to 6.74)**	2.2	**3.53 (1.63 to 7.64)**
Score of frequency			**1.33 (1.25 to 1.41)**		**1.36 (1.23 to 1.51)**		**1.43 (1.23 to 1.65)**		**1.53 (1.22 to 1.93)**

Note: % refers to percent of positive ideation, plans, preparation and attempts in each type of school bullying experience

^a^ Model adjusted for gender, self-estimated family economic status, relationship with mother, relationship with father and number of friends that were statistically significant in univariate analyses

^b^ Model adjusted for gender, relationship with mother, relationship with father and number of friends

^c^ Model adjusted for gender and number of friends

^d^ Model adjusted for gender

*aOR* Adjusted odds ratios, *CI* Confidence interval

Variable levels significant at *p* < 0.05 are in boldface type

### Role in school bullying and murderous ideation and Behaviours

Table 5 shows the association between the roles that adolescents played in school bullying and the murder-related outcomes. This analysis tested the third hypothesis that the roles of bully, victim and bully-victim have different associations with murderous ideation and behaviours. We divided the study participants into three subgroups based on their roles (i.e., bully only, victim only, bully-victim) in school bullying. Compared with those who had no bully experience, bullies had significantly higher ORs for murderous ideation [4.04 (2.94 to 5.55)], plans [3.79 (2.17 to 6.63)], preparation [3.84 (1.74 to 8.47)]; victims had significantly higher odds for ideation [2.10 (1.68 to 2.63)] and plans [1.60 (1.02 to 2.51)]; and bully-victims had significantly higher odds for ideation [3.88 (3.07 to 4.90)], plans [4.69 (3.15 to 6.98)], preparation [5.99 (3.45 to 10.41)], and attempts [7.24 (3.08 to 17.02)].

**Table 5:** Multivariate logistic regression of adolescent murderous ideation and behaviours on role in school bullying (*N* = 5726)

Role of school bullying	%	Ideation		Plans		Preparation		Attempts	
		%	aOR (95%CI) ^a^	%	aOR (95%CI) ^b^	%	aOR (95%CI) ^c^	%	aOR (95%CI) ^d^
Non-involved	59.3	5.7	1.00 [Ref]	1.4	1.00 [Ref]	0.6	1.00 [Ref]	0.2	1.00 [Ref]
Bully only	22.2	20.5	**4.04 (2.94 to 5.55)**	**5.9**	**3.79 (2.17 to 6.63)**	**2.9**	**3.84 (1.74 to 8.47)**	1.0	3.24 (0.85 to 12.34)
Victim only	5.4	12.1	**2.10 (1.68 to 2.63)**	**2.6**	**1.60 (1.02 to 2.51)**	0.9	1.25 (0.60 to 2.59)	0.3	1.15 (0.35 to 3.85)
Bully-victim	13.1	21.1	**3.88 (3.07 to 4.90)**	**7.9**	**4.69 (3.15 to 6.98)**	4.5	**5.99 (3.45 to 10.41)**	**2.3**	**7.24 (3.08 to 17.02)**

Note: % refers to percent of positive ideation, plans, preparation and attempts in each type of school bullying experience

^a^ Model adjusted for gender, self-estimated family economic status, relationship with mother, relationship with father and number of friends that were statistically significant in univariate analyses

^b^ Model adjusted for gender, relationship with mother, relationship with father and number of friends

^c^ Model adjusted for gender and number of friends

^d^ Model adjusted for gender

aOR Adjusted odds ratios, *CI* Confidence interval

Variable levels significant at *p* < 0.05 are in boldface type

## Discussion

To our knowledge, this study is the first to examine the association between school bullying and murderous ideation and behaviours in China. The study results indicate that involvement in school bullying is associated with higher odds of murderous ideation and behaviours. The odds of murderous ideation and behaviours increase with the number of bullying types and the frequency of bullying, and the relevant dose-response relationships are found in both bullies and victims. Our findings indicate that males, those with low socioeconomic status (SES), and those with poor family relationships have increased odds of murderous ideation and behaviours, and these findings are consistent with those of previous studies [18–21, 38].

Our results support the first assumption that both adolescents who perpetrated and adolescents who experienced traditional and cyber bullying have higher odds of murderous ideation and behaviours. Cyber bullying has received less attention from the public; however, it has occurred more frequently in the last decade due to increases in Internet and cell phone use among adolescents [31, 39, 40]. Compared to traditional bullying, cyber bullying can spread quickly, affect a large number of adolescents, and be difficult to trace back to the source, and it is not limited by time and location and usually involves a lack supervision [31, 39, 40]. Moreover, we found extremely high ORs (> 20) between cyber bullying and murderous attempts. With the rapid increase of adolescent users on the Internet or wireless networks, our findings highlight the need to pay attention to cyber bullying in order to prevent adolescent murders.

Both bullies and victims have increased odds of all four types of murderous ideation and behaviours, and these associations are amplified in those who both perpetrate bullying and experience bullying. Previous studies have suggested that adolescents who are involved in bullying have deficiencies in social information processing patterns [28, 41]. Both bullies and victims tend to interpret others’ languages and behaviours as hostile, and bully-victims tend to act like bullies rather than victims [41]. Further, victims may carry weapons for self-protection, whereas bullies may carry weapons to intimidate others [42]. Studies have also found that bully-victims are at the highest risk of carrying weapons compared with bullies or victims [43, 44]. However, the underlying mechanisms of the role played in bullying and murderous ideation and behaviours are complex. We will explore these mechanisms in the future.

Our findings also suggest dose-response relationships between types and frequencies of school bullying and murderous ideation and behaviours. Previous studies have shown an association between the frequency of bullying and the risk of increased depression and poor sleep quality [32, 33]. Holt [45] found that urban elementary students who experienced multiple types of victimization were at greater risk for psychological distress and lower academic performance. Our results further extend previous findings indicating that experiencing a higher frequency of bullying and experiencing multiple types of bullying have more adverse health impacts on adolescents.

Our study found a positive link between school bullying and adolescent murderous ideation and behaviours. This finding is partly supported by a previous study that found that involvement in bullying as a victim, bully, or bully-victim is related to weapon carrying [24]. However, due to the high prevalence of school bullying and the rare cases of homicide among adolescents, bullying was not considered a causal factor of adolescent murder [46, 47]. Nevertheless, we cannot conclude that there is a causal relationship between school bullying and murderous ideation and behaviours in this study due to its cross-sectional design. In addition, cultural differences might influence the relationship between school bullying and murderous ideation and behaviours. In the United States, a murder attempt often results in death due to the use of guns [47]. In China, guns are regulated, and murderous attacks have a low rate of death. Therefore, adolescent murder may be under-reported in China because social media tend to report only murder attempts that result in death. Additionally, China has a different culture and different beliefs than Western countries, and Chinese cultures vary greatly within the country. Future studies will investigate the role of cultural differences in school bullying and adolescent murders.

Among the four types of murder-related study outcomes we investigated, murderous attempts generally exhibited the highest ORs for each type of bullying experienced. Meanwhile, murderous attempts represent the most serious murder-related ideation and behaviours investigated in this study. The results indicated that school bullying has a greater impact on more serious murder-related outcomes. However, by comparing this result between adolescents who committed bullying and adolescents who experienced bullying, we found that the associations among the four murder-related study outcomes varied greatly for bullies, while the variation was not as high for victims. Generally, bullies’ ORs for all four bullying types increased with the severity of murder-related study outcomes. However, the ORs for victims were less than the corresponding ORs observed for bullies, and no clear trend was found regarding the severity of murder-related outcomes. However, these findings are best regarded as preliminary. Future studies are needed to confirm our results and explore the mechanisms underlying the association between school bullying and murderous ideation and behaviours.

### Limitations

Although our study has many advantages, such as the adoption of random sampling approach and a large sample size, our study design has a few limitations. First, this was a cross-sectional study, and the survey periods for school bullying and murder were not synchronous; therefore, no causal relationship can be established from our study results. Second, the data we collected are vulnerable to recall bias. The students were surveyed about their experiences with school bullying in the last two months to reduce recall bias, but the participants were asked to report their murderous ideation and behaviours in the past six months to enhance the reporting rates. On the one hand, murderous ideation and behaviours represent a type of rare psychological behaviour and may be easy to recall. On the other hand, adolescent bullying often repeats over time, and the rate remains quite stable over time; thus, the frequency of bullying in the last two months might be close to that in the past six months. In the future, a cohort study design should be applied to explore the causal relationship between school bullying and adolescent murder and behaviours. Further, participants might under-report their murderous ideation and behaviours. A method combining register-based and self-reported data sources is needed to better understand the true rate of murderous ideation and behaviours among adolescents. Third, we did not include other potential risk factors for murderous ideation and behaviours in this study, such as character, self-esteem, mental health, and problem-solving ability.

## Conclusion

In this study, school bullying was found to be associated with increased risk for adolescent murderous ideation and behaviours. There was a dose-response association between the frequency of school bullying and the number of bullying types and murderous ideation and behaviours. Our results indicate there is a link between school bullying and murderous ideation and behaviours in Chinese adolescents. The study results can provide a reference for plans to prevent and intervene in adolescent murder.

## Additional files

Additional file 1: **Table S1.** Multi-level logistic regression between individual type of school bullying and adolescent murderous ideation and behaviours (*N* = 5726). Results of two-level logistic regression mixed models in which classrooms were treated as clusters using the package “lme4” in R version 3.5.1 to confirm the relationships between individual type of school bullying and adolescent murderous ideation and behaviours, with adjustments for sociodemographic variables. (DOC 48 kb)

Additional file 2: **Table S2.** Multi-level logistic regression of adolescent murderous ideation and behaviours on number of school bullying types (*N* = 5726). Results of two-level logistic regression mixed models to confirm the relationships between number of school bullying types and adolescent murderous ideation and behaviours, with adjustments for sociodemographic variables. (DOC 67 kb)

Additional file 3: **Table S3.** Multi-level logistic regression of adolescent murderous ideation and behaviours on frequency of school bullying (*N* = 5726). Results of two-level logistic regression mixed models to confirm the relationships between frequency of school bullying and adolescent murderous ideation and behaviours, with adjustments for sociodemographic variables. (DOC 63 kb)

Additional file 4: **Table S4.** Multi-level logistic regression of adolescent murderous ideation and behaviours on role in school bullying (*N* = 5726). Results of two-level logistic regression mixed models to confirm the relationships between role in school bullying and adolescent murderous ideation and behaviours, with adjustments for sociodemographic variables. (DOC 42 kb)

## Abbreviations

aOR: Adjusted Odds Ratio
SD: Standard Deviation
SPSS: Statistical Package of Social Sciences and Problem Solutions
