# Supplementary material for: Is involvement in school bullying associated with increased risk of murderous ideation and behaviours among adolescent students in China?
Source: BMC Psychiatry. 2019 Apr 24;19:121. doi: 10.1186/s12888-019-2108-5 (PMC6480810; doi:10.1186/s12888-019-2108-5)
Supplement: Supplementary file 1 — Table S1. Multi-level logistic regression between individual type of school bullying and adolescent murderous ideation and behaviours (N = 5726). Results of two-level logistic regression mixed models in which classrooms were treated as clusters using the package “lme4” in R version 3.5.1 to confirm the relationships between individual type of school bullying and adolescent murderous ideation and behaviours, with adjustments for sociodemographic variables. (DOC 48 kb) [file 12888_2019_2108_MOESM1_ESM.doc]

**Table** **S1** Multi-level logistic regression between individual type of school bullying and adolescent murderous ideation and behaviours (*N*=5726)

| Type of school bullying experience | % | Ideation | |  | Plans | |  | Preparation | |  | Attempts | |
| --- | --- | --- | --- | --- | --- | --- | --- | --- | --- | --- | --- | --- |
|  |  | % | aOR (95%CI) ^a^ |  | % | aOR (95%CI) ^b^ |  | % | aOR (95%CI) ^c^ |  | % | aOR (95%CI) ^d^ |
| Bully |  |  |  |  |  |  |  |  |  |  |  |  |
| Physical (yes vs no) | 3.5 | 23.2 | **2.34(1.63 to 3.31)** |  | 15.2 | **5.64(3.58 to 8.66)** |  | 9.6 | **7.91(4.42 to 13.59)** |  | 6.1 | **12.22(5.61 to 25.62)** |
| Verbal (yes vs no) | 6.6 | 24.2 | **2.77(2.12 to 3.58)** |  | 11.1 | **4.25(2.87 to 6.19)** |  | 6.1 | **5.02(2.93 to 8.32)** |  | 3.9 | **8.84(4.23 to 18.34)** |
| Relational (yes vs no) | 3.8 | 25.8 | **3.06(2.19 to 4.21)** |  | 16.6 | **7.37(4.83 to 11.00)** |  | 10.6 | **10.72(6.24 to 17.80)** |  | 5.1 | **10.83(4.90 to 22.71)** |
| Cyber (yes vs no) | 2.4 | 24.3 | **2.51(1.64 to 3.75)** |  | 17.6 | **6.53(3.93 to 10.48)** |  | 14.7 | **13.35(7.47 to 22.94)** |  | 10.3 | **24.89(11.58 to 52.53)** |
| Victim |  |  |  |  |  |  |  |  |  |  |  |  |
| Physical (yes vs no) | 6.1 | 20.5 | **2.12(1.58 to 2.82)** |  | 8.4 | **2.75(1.76 to 4.18)** |  | 4.6 | **3.36(1.83 to 5.84)** |  | 3.2 | **5.87(2.67 to 12.28)** |
| Verbal (yes vs no) | 15.7 | 15.9 | **1.67(1.36 to 2.09)** |  | 5.7 | **2.08(1.45 to 2.94)** |  | 2.9 | **2.37(1.43 to 3.83)** |  | 1.6 | **3.15(1.51 to 6.41)** |
| Relational (yes vs no) | 10.8 | 19.7 | **2.34(1.86 to 2.93)** |  | 7.3 | **3.09(2.13 to 4.42)** |  | 3.6 | **3.50(2.06 to 5.73)** |  | 1.8 | **4.16(1.92 to 8.56)** |
| Cyber (yes vs no) | 2.7 | 22.7 | **2.38(1.58 to 3.50)** |  | 14.3 | **5.28(3.15 to 8.50)** |  | 9.1 | **7.21(3.73 to 13.10)** |  | 9.1 | **23.14(10.83 to 48.46)** |

Note: % refers to percent of positive ideation, plans, preparation and attempts in each type of school bullying experience.

**^a^** Two-level logistic regression mixed models in which classrooms were treated as clusters adjusted for gender, self-estimated family economic status, relationship with mother, relationship with father and number of friends that were statistically significant in univariate analyses.

**^b^** Two-level logistic regression mixed models in which classrooms were treated as clusters adjusted for gender, relationship with mother, relationship with father and number of friends.

**^c^** Two-level logistic regression mixed models in which classrooms were treated as clusters adjusted for gender and number of friends.

**^d^** Two-level logistic regression mixed models in which classrooms were treated as clusters adjusted for gender.

aOR - adjusted odds ratios; CI - confidence interval.

Variable levels significant at *p* < 0.05 are in **boldface type**.
