# Supplementary material for: Is involvement in school bullying associated with increased risk of murderous ideation and behaviours among adolescent students in China?
Source: BMC Psychiatry. 2019 Apr 24;19:121. doi: 10.1186/s12888-019-2108-5 (PMC6480810; doi:10.1186/s12888-019-2108-5)
Supplement: Supplementary file 2 — Table S2. Multi-level logistic regression of adolescent murderous ideation and behaviours on number of school bullying types (N = 5726). Results of two-level logistic regression mixed models to confirm the relationships between number of school bullying types and adolescent murderous ideation and behaviours, with adjustments for sociodemographic variables. (DOC 67 kb) [file 12888_2019_2108_MOESM2_ESM.doc]

**Table S2** Multi-level logistic regression of adolescent murderous ideation and behaviours on number of school bullying types (*N*=5726)

| Variety of school bullying | % | Ideation | |  | Plans | |  | Preparation | |  | Attempts | |
| --- | --- | --- | --- | --- | --- | --- | --- | --- | --- | --- | --- | --- |
|  |  | % | aOR (95%CI) ^a^ |  | % | aOR (95%CI) ^b^ |  | % | aOR (95%CI) ^c^ |  | % | aOR (95%CI) ^d^ |
| Bully |  |  |  |  |  |  |  |  |  |  |  |  |
| Non-involved | 76.7 | 8.4 | 1.00 [Ref] |  | 1.9 | 1.00 [Ref] |  | 0.8 | 1.00 [Ref] |  | 0.3 | 1.00 [Ref] |
| One type | 15.2 | 22.5 | **2.86 (2.18 to 3.72)** |  | 7.1 | **3.27(2.05 to 5.04)** |  | 3.1 | **3.49(1.73 to 6.53)** |  | 1.3 | **4.18(1.31 to 11.26)** |
| Two types | 5.0 | 22.0 | **2.53(1.54 to 4.01)** |  | 10.1 | **4.42(2.15 to 8.29)** |  | 5.5 | **5.66(2.10 to 12.87)** |  | 2.8 | **7.67(1.73 to 24.74)** |
| Three types | 2.2 | 22.4 | **2.62(1.25 to 5.07)** |  | 18.4 | **9.25(4.04 to 19.23)** |  | 14.3 | **15.60(6.05 to 35.42)** |  | 6.1 | **17.65(3.87 to 58.67)** |
| Four types | 0.9 | 32.6 | **4.26(2.19 to 7.95)** |  | 30.4 | **16.93(8.32 to 32.95)** |  | 21.7 | **28.38(12.43 to 60.41)** |  | 17.4 | **58.77(21.36 to 152.24)** |
| Number of types |  |  | **1.57 (1.40 to 1.75)** |  |  | **2.08(1.81 to 2.39)** |  |  | **2.36(1.99 to 2.78)** |  |  | **2.71(2.15 to 3.39)** |
| Victim |  |  |  |  |  |  |  |  |  |  |  |  |
| Non-involved | 89.7 | 8.0 | 1.00 [Ref] |  | 2.0 | 1.00 [Ref] |  | 0.9 | 1.00 [Ref] |  | 0.3 | 1.00 [Ref] |
| One type | 6.7 | 13.4 | **1.60 (1.27 to 2.00)** |  | 3.4 | 1.52(0.98 to 2.31) |  | 1.5 | 1.50(0.76 to 2.75) |  | 0.6 | 1.78(0.56 to 4.84) |
| Two types | 1.9 | 19.9 | **2.50(1.81 to 3.43)** |  | 7.0 | **3.03(1.77 to 4.97)** |  | 3.1 | **3.15(1.41 to 6.33)** |  | 1.4 | **4.07(1.12 to 11.90)** |
| Three types | 0.9 | 25.8 | **3.53(2.27 to 5.39)** |  | 12.1 | **5.14(2.73 to 9.11)** |  | 7.3 | **6.98(3.07 to 14.37)** |  | 5.6 | **15.95(5.73 to 41.42)** |
| Four types | 0.8 | 21.6 | **2.47(1.18 to 4.78)** |  | 15.7 | **6.93(2.90 to 14.73)** |  | 9.8 | **8.94(2.93 to 22.39)** |  | 7.8 | **19.70(5.23 to 60.84)** |
| Number of types |  |  | **1.46 (1.33 to 1.60)** |  |  | **1.68(1.46 to 1.93)** |  |  | **1.80(1.49 to 2.16)** |  |  | **2.26(1.76 to 2.89)** |

Note: % refers to percent of positive ideation, plans, preparation and attempts in each type of school bullying experience.

**^a^** Two-level logistic regression mixed models in which classrooms were treated as clusters adjusted for gender, self-estimated family economic status, relationship with mother, relationship with father and number of friends that were statistically significant in univariate analyses.

**^b^** Two-level logistic regression mixed models in which classrooms were treated as clusters adjusted for gender, relationship with mother, relationship with father and number of friends.

**^c^** Two-level logistic regression mixed models in which classrooms were treated as clusters adjusted for gender and number of friends.

**^d^** Two-level logistic regression mixed models in which classrooms were treated as clusters adjusted for gender.

aOR - adjusted odds ratios; CI - confidence interval.

Variable levels significant at *p* < 0.05 are in **boldface type**.
