# Supplementary material for: Is involvement in school bullying associated with increased risk of murderous ideation and behaviours among adolescent students in China?
Source: BMC Psychiatry. 2019 Apr 24;19:121. doi: 10.1186/s12888-019-2108-5 (PMC6480810; doi:10.1186/s12888-019-2108-5)
Supplement: Supplementary file 3 — Table S3. Multi-level logistic regression of adolescent murderous ideation and behaviours on frequency of school bullying (N = 5726). Results of two-level logistic regression mixed models to confirm the relationships between frequency of school bullying and adolescent murderous ideation and behaviours, with adjustments for sociodemographic variables. (DOC 63 kb) [file 12888_2019_2108_MOESM3_ESM.doc]

**Table S3** Multi-level logistic regression of adolescent murderous ideation and behaviours on frequency of school bullying (*N*=5726)

| Frequency of school bullying | % | Ideation | |  | Plans | |  | Preparation | |  | Attempts | |
| --- | --- | --- | --- | --- | --- | --- | --- | --- | --- | --- | --- | --- |
|  |  | % | aOR (95%CI) ^a^ |  | % | aOR (95%CI) ^b^ |  | % | aOR (95%CI) ^c^ |  | % | aOR (95%CI) ^d^ |
| Bully |  |  |  |  |  |  |  |  |  |  |  |  |
| Non-involved | 62.3 | 6.3 | 1 [Reference] |  | 1.7 | 1 [Reference] |  | 0.7 | 1 [Reference] |  | 0.3 | 1 [Reference] |
| Less than twice a month | 19.3 | 11.0 | **1.80(1.42 to 2.27)** |  | 1.9 | 1.04(0.61 to 1.69) |  | 0.6 | 0.79(0.31 to 1.73) |  | 0.1 | 0.25(0.01 to 1.32) |
| Two or three times a month | 8.1 | 16.5 | **2.83(2.12 to 3.75)** |  | 3.2 | 1.66(0.90 to 2.89) |  | 1.3 | 1.56(0.58 to 3.57) |  | 0.2 | 0.57(0.03 to 2.96) |
| Once a week | 4.8 | 26.2 | **4.72(3.45 to 6.41)** |  | 6.9 | **3.50(1.99 to 5.89)** |  | 3.6 | **4.32(1.95 to 8.88)** |  | 0.7 | 1.76(0.27 to 6.68)) |
| Several times a week or more | 5.5 | 23.2 | **3.88(2.84 to 5.25)** |  | 13.7 | **6.95(4.51 to 10.64)** |  | 8.6 | **9.78(5.52 to 17.40)** |  | 5.4 | **12.00(5.47 to 27.44)** |
| Score of frequency |  |  | **1.49(1.39 to 1.58)** |  |  | **1.62(1.46 to 1.81)** |  |  | **1.82(1.56 to 2.11)** |  |  | **1.98(1.58 to 2.50)** |
| Victim |  |  |  |  |  |  |  |  |  |  |  |  |
| Non-involved | 42.1 | 6.2 | 1 [Reference] |  | 1.7 | 1 [Reference] |  | 0.9 | 1 [Reference] |  | 0.5 | 1 [Reference] |
| Less than twice a month | 22.6 | 8.3 | **1.32(1.01 to 1.71)** |  | 1.9 | 1.05(0.63 to 1.73) |  | 0.8 | **0.85(0.38 to 1.83)** |  | 0.0 | NA |
| Two or three times a month | 13.2 | 12.1 | **1.99(1.50 to 2.62)** |  | 2.4 | 1.25(0.69 to 2.15) |  | 0.9 | **0.99(0.40 to 2.37)** |  | 0.1 | 0.25(0.03 to 2.01) |
| Once a week or more | 8.4 | 13.8 | **2.12(1.54 to 2.89)** |  | 3.8 | 1.77(0.98 to 3.08) |  | 1.7 | **1.68(0.71 to 3.89)** |  | 0.6 | 1.06(0.29 to 3.99) |
| Several times a week or more | 13.8 | 19.7 | **3.30(2.57 to 4.25)** |  | 7.1 | **3.37(2.12 to 5.17)** |  | 3.8 | **3.80(2.10 to 6.77)** |  | 2.2 | **3.53(1.60 to 7.76)** |
| Score of frequency |  |  | **1.34(1.26 to 1.42)** |  |  | **1.36(1.23 to 1.51)** |  |  | **1.43(1.23 to 1.66)** |  |  | **1.53(1.22 to 1.94)** |

Note: % refers to percent of positive ideation, plans, preparation and attempts in each type of school bullying experience.

**^a^** Two-level logistic regression mixed models in which classrooms were treated as clusters adjusted for gender, self-estimated family economic status, relationship with mother, relationship with father and number of friends that were statistically significant in univariate analyses.

**^b^** Two-level logistic regression mixed models in which classrooms were treated as clusters adjusted for gender, relationship with mother, relationship with father and number of friends.

**^c^** Two-level logistic regression mixed models in which classrooms were treated as clusters adjusted for gender and number of friends.

**^d^** Two-level logistic regression mixed models in which classrooms were treated as clusters adjusted for gender.

aOR - adjusted odds ratios; CI - confidence interval.

Variable levels significant at *p* < 0.05 are in **boldface type**.
