# Supplementary material for: Is involvement in school bullying associated with increased risk of murderous ideation and behaviours among adolescent students in China?
Source: BMC Psychiatry. 2019 Apr 24;19:121. doi: 10.1186/s12888-019-2108-5 (PMC6480810; doi:10.1186/s12888-019-2108-5)
Supplement: Supplementary file 4 — Table S4. Multi-level logistic regression of adolescent murderous ideation and behaviours on role in school bullying (N = 5726). Results of two-level logistic regression mixed models to confirm the relationships between role in school bullying and adolescent murderous ideation and behaviours, with adjustments for sociodemographic variables. (DOC 42 kb) [file 12888_2019_2108_MOESM4_ESM.doc]

**Table S4** Multi-level logistic regression of adolescent murderous ideation and behaviours on role in school bullying (*N*=5726)

| Role of school bullying | % | Ideation | |  | Plans | |  | Preparation | |  | Attempts | |
| --- | --- | --- | --- | --- | --- | --- | --- | --- | --- | --- | --- | --- |
|  |  | % | aOR (95%CI) ^a^ |  | % | aOR (95%CI) ^b^ |  | % | aOR (95%CI) ^c^ |  | % | aOR (95%CI) ^d^ |
| Non-involved | 59.3 | 5.7 | 1.00 [Ref] |  | 1.4 | 1.00 [Ref] |  | 0.6 | 1.00 [Ref] |  | 0.2 | 1.00 [Ref] |
| Bully only | 22.2 | 20.5 | **3.98(2.88 to 5.45)** |  | **5.9** | **3.65(1.01 to 2.51)** |  | **2.9** | **3.81(1.64 to 8.18)** |  | 1.0 | 3.10(0.67 to 10.89) |
| Victim only | 5.4 | 12.1 | **2.13(1.70 to 2.67)** |  | **2.6** | **1.61(2.03 to 6.29)** |  | 0.9 | 1.25(0.58 to 2.54) |  | 0.3 | 1.16(0.31 to 3.70) |
| Bully-victim | 13.1 | 21.1 | **4.02(3.16 to 5.09)** |  | **7.9** | **4.58(3.07 to 6.88)** |  | 4.5 | **5.97(3.45 to 10.52)** |  | **2.3** | **7.06(3.07 to 17.61)** |

Note: % refers to percent of positive ideation, plans, preparation and attempts in each type of school bullying experience.

**^a^** Two-level logistic regression mixed models in which classrooms were treated as clusters adjusted for gender, self-estimated family economic status, relationship with mother, relationship with father and number of friends that were statistically significant in univariate analyses.

**^b^** Two-level logistic regression mixed models in which classrooms were treated as clusters adjusted for gender, relationship with mother, relationship with father and number of friends.

**^c^** Two-level logistic regression mixed models in which classrooms were treated as clusters adjusted for gender and number of friends.

**^d^** Two-level logistic regression mixed models in which classrooms were treated as clusters adjusted for gender.

aOR- adjusted odds ratios; CI - confidence interval.

Variable levels significant at *p* < 0.05 are in **boldface type**.
